# Considerations for the Use of Polyphenols as Therapies in Neurodegenerative Diseases

**DOI:** 10.3390/ijms20081883

**Published:** 2019-04-16

**Authors:** Justine Renaud, Maria-Grazia Martinoli

**Affiliations:** 1 Cellular Neurobiology, Department of Medical Biology, Université du Québec, Trois-Rivières, Québec, QC G9A5H7, Canada; justine.renaud@uqtr.ca; 2 Department of Psychiatry & Neuroscience, Université Laval and CHU Research Center, Ste-Foy, QC G1V 4G2, Canada

**Keywords:** neurodegeneration, neuroprotection, nutraceuticals, bioavailability, stress response

## Abstract

Over the last two decades, the increase in the incidence of neurodegenerative diseases due to the increasingly ageing population has resulted in a major social and economic burden. At present, a large body of literature supports the potential use of functional nutrients, which exhibit potential neuroprotective properties to mitigate these diseases. Among the most studied dietary molecules, polyphenols stand out because of their multiple and often overlapping reported modes of action. However, ambiguity still exists as to the significance of their influence on human health. This review discusses the characteristics and functions of polyphenols that shape their potential therapeutic actions in neurodegenerative diseases while the less-explored gaps in knowledge of these nutrients will also be highlighted.

## 1. Introduction

It is widely acknowledged that nutrition plays a key role in the occurrence and progression of non-communicable diseases. A body of epidemiological evidence shows that a diet rich in fruit and vegetables reduces the incidence of cardiovascular diseases [1,2,3,4], type 2 diabetes [5,6], stroke [7,8] and numerous cancers [9,10,11]. Other studies find an inverse association between the consumption of green tea and cognitive decline [12,13]. These observed health benefits are thought to be at least partly attributable to a class of non-essential nutrients named polyphenols, found abundantly in fruits and vegetables [14,15].

Together with cancer and cardiovascular diseases, neurodegenerative disorders constitute a potential application for the benefits of polyphenols [16,17]. This includes Parkinson’s and Alzheimer’s diseases which lack clear etiopathogenetic origins and arise from the interaction between aging, environment and genetic risk factors. The etiology of these diseases is further complicated by a number of proposed causative mechanisms, including oxidative stress, neuroinflammation, protein aggregation, iron toxicity and mitochondrial dysfunction. Polyphenols are reported to improve many of these factors at a cellular level, which makes their use in complex neurodegenerative disorders compelling. In this review, the properties that may influence the functionality and bioavailability of dietary polyphenols in the central nervous system (CNS) are discussed with a particular focus on therapeutic applications and limitations.

## 2. Chemico-Structural Characteristics

### 2.1. Classification

Plant polyphenols were originally classified in the early literature as “vegetable tannins” owing to their tanning action on animal skins [18]. The first comprehensive description, referred to as the White–Bate-Smith–Swain–Haslam (WBSSH) definition, recommended that the term polyphenol be exclusively used to describe water-soluble phenolic compounds having a molecular mass ranging between 500 to 4000 Da, possessing at least 12 phenolic hydroxyl groups and 5 to 7 aromatic rings per 1000 Da [19]. A less restrictive interpretation was proposed offering a broader view of the WBSSH definition to include simpler phenolic compounds with potential biological activities others than tanning [20]:

“The term “polyphenol” should be used to define compounds exclusively derived from the shikimate/phenylpropanoid and/or the polyketide pathway, featuring more than one phenolic unit and deprived of nitrogen-based functions. This definition lets out all monophenolic structures as well as all their naturally occurring derivatives such as phenyl esters, methyl phenyl ethers and O-phenyl glycosides.”

A majority of plant polyphenols originate from phenylalanine which is deaminated to cinnamic acid, which then enters the phenylpropanoid pathway [21]. Plant metabolism utilizes the phenylpropanoid unit C6-C3, a phenol ring with a 3-carbon side chain, as a building block to construct polyphenols. Classification of the resulting molecules is dictated by the number of phenol rings (C6) they contain and the structural elements binding these rings to one another. The main subclasses, varying in complexity, are phenolic acids (C6-C3 and C6-C1), flavonoids (C6-C3-C6), stilbenes (C6-C2-C6) and lignans (C6-C3-C3-C6). Within these subclasses, hydroxylations and O-glycosylations at various positions as well as *cis-trans* isomerization give rise to the thousands of polyphenols (estimated to be >8000) identified to date, resulting in a complex range of molecules with potential pharmacological values. Details of these polyphenols alongside their occurrence in various food products are available on databases such as Phenol-Explorer managed by the Institut National de la Recherche Agronomique (www.phenol-explorer.eu).

### 2.2. Structure versus Biofunctionality in Neuroprotection

The structural properties shared by polyphenols are important to their potential therapeutic applications, particularly in neuroprotection. These include the presence of phenol rings, variable hydroxylation patterns and conjugated double bonds all of which grant polyphenols metal-chelating, fibril-destabilizing, estrogen-like, enzyme-binding and antioxidative properties. These modes of action allow polyphenols to provide a defense against many pathophysiological aspects of neurodegenerative diseases, namely oxidative stress, neuroinflammation, protein aggregation, iron toxicity and mitochondrial dysfunction. These are detailed below:

The redox properties of divalent metals, such as copper, zinc and iron, are essential for cellular homeostasis. When in excess, however, these metals generate surplus reactive oxygen species. This excess can be reversed by chelation with polyphenols that possess at least one galloyl or catechol group (hydroxyl groups in the *ortho*-position) which are powerful bidentate chelators of divalent metals [22], whereas polyphenols having only a phenol substitution (one hydroxyl function) or possessing a resorcinol group (*meta*-position hydroxyl pair) are less potent monodentate chelators [23,24]. For chelation to occur, a deprotonation step of the phenolic group is necessary and has been shown to be possible at physiological pH [23].

Self-assembly of amyloidogenic fibrils including tau, beta amyloid (Aβ) and α-synuclein all neuropathologically relevant proteins involves interactions between aromatic residues [25]. Using similar aromatic interactions, as described above, phenol moieties in polyphenols can interfere with fibril assembly [26], possibly by weakening cross-β structures. This interference seems to arise from hydrophobic and π stacking interactions [27], although the formation of covalent bonds through Schiff base reactions has also been proposed for the green tea polyphenol epigallocatechin-3-gallate (EGCG) [28,29]. Analysis of binding energies between polyphenols and protein fibrils has also shown favorable entropic and enthalpic dynamics that suggest the stabilization of H-bonds [30].

Polyphenols, referred to as phytoestrogens, have the ability to bind estrogen receptors (ERs), usually with a greater affinity for ERβ [31,32]. Depending on structure, dose, cell type and estrogen response element (ERE) sequence, different polyphenols have a weak or strong antagonistic or agonistic effect on ERs, resulting in a wide spectrum of activities in cells [33,34,35,36]. To enable binding to ERs, a structure should be composed of a phenolic ring with a configuration resembling that of estradiol, as found in flavonoid isoflavones or the stilbene resveratrol, for instance. Also, a specific hydroxylation pattern and an adequate distance between substituted hydroxyl groups are necessary to bind ERs.

Polyphenols can also share structural similarities with endogenous ligands, such as cyclic adenosine monophosphate (cAMP) or nucleoside triphosphates, endowing them with the aptitude to activate or inhibit key enzymes [37,38]. To date, the modulatory effects of several polyphenols on enzymes have been confirmed in cellular or animal models, these include resveratrol on cAMP phosphodiesterases [39], theaflavins on the adenosine triphosphate (ATP) synthase and respiratory chain [40] and curcumin on glyoxalase 1 [41]. The presence of appropriately spaced ketone and hydroxyl groups in a planar configuration, bestow some polyphenols, such as curcumin, with the ability to mimic an enediolate intermediate in physiological conditions [42] is an example of structural elements that make enzyme binding possible.

Apart from the functions described above which result from the unique chemical structures of polyphenols, the most vastly studied characteristic of this class of chemicals is their antioxidative action. Polyphenols are thought to exert their antioxidative action directly, by scavenging free radical species firsthand, and/or indirectly, by activating endogenous antioxidative pathways. Direct antioxidative effects usually occur through H-atom transfer from polyphenols’ (ArOH) hydroxyl (OH) groups to the free radicals (R•):

ArOH + R• → ArO• + RH(1)

The existence of multiple conjugated double bonds in polyphenols allows unpaired electron to be delocalized over the aromatic ring, yielding a much more stable and much less reactive, polyphenolic radical (ArO•) (Equation (1)). Some polyphenols also exert indirect antioxidative effects through the Kelch-like ECH-associated protein 1/nuclear factor erythroid 2-related factor 2/antioxidant response elements (Keap1/Nrf2/ARE) regulatory pathway made possible by the presence of electrophilic functions (α,β-unsaturated carbonyl group, 1,2- and 1,4-quinones or other groups) that alkylate thiol sensors in the cysteine pocket of Keap1 [43,44]. Others, like stilbenes, engage their resorcinol hydroxyl functions in hydrogen bonds with the Kelch pocket of Keap1 [45]. Both these events lead to the disruption of the Keap1/Nrf2 complex, allowing Nrf2 to translocate to the nucleus where it can trigger the expression of antioxidant proteins like heme oxygenase-1 via binding of adenylate and uridylate (AU)-rich elements (AREs). This cysteine-modifying function of polyphenols may also have implications for the activity of various other enzymes [44].

## 3. Factors Influencing Pharmacokinetics and Bioavailability

To be effective in the prevention or amelioration of neurodegenerative diseases, polyphenols must be bioavailable. Extensive reports on the bioavailability of the most common dietary polyphenols can be found elsewhere [46,47,48]. In this review, we will first discuss the obstacles that hinder polyphenol bioavailability and address CNS permeability in particular.

### 3.1. Food Matrix or Vehicle

Oral administration is the most usual route if polyphenols are given pharmacologically but this often conflicts with bioavailability. Particular factors include interaction with vehicle, transformations by digestive and microbial enzymes and absorption by the gastrointestinal tract [49].

Food matrices are central to the efficacy of polyphenols [50]. Few studies have been conducted and inconsistent results have been obtained, demonstrating either a negligible [51,52] or a significant [53,54,55,56] contribution of the food matrix to polyphenol absorption. Indeed, peculiar factors such as the type of lipid matrix used may mediate in the release of polyphenols in the gastrointestinal tract [57,58]. Ethanol may also play a role in polyphenols absorption with studies showing improved bioavailability of quercetin in rats when administered in 30% ethanol, an alcohol content that is unsustainable in the diet [59]. In humans administered normal or dealcoholized red wine there was no differences in plasma levels of catechin but increased catechin excretion with red wine probably due to a diuretic effect of alcohol [60]. However, matrix effects are too peculiar to be fully reviewed here.

### 3.2. Gastrointestinal Transformations and Absorption

Absorption and metabolism of polyphenols have extensively studied (see for review, References [61,62]). Whereas aglycones are normally well absorbed by the small intestine, nutritional polyphenols are more commonly present as glycosides, esters and polymers, which cannot be efficiently assimilated in the upper portion of the gut.

Molecules not absorbed in the upper gastrointestinal tract continue to the colon to become substrates for the gut microbiota, responsible for a very wide array of reactions, some of which yield monomers or aglycones from glycosylated polyphenols (see for review [63]). Smaller, better-absorbed phenolic acids may also be produced by the gut microbiota. For example, microbiotic degradation of quercetin mainly generates 3,4-dihydroxyphenylacetic, 3-methoxy-4-hydroxyphenylacetic (homovanillic acid) and 3-hydroxyphenylacetic acid [64]. In volunteers challenged with 75 mg of rutin, a quercetin glycoside, the total urinary excretion of microbial metabolites accounted for as much as 50% of the ingested dose [65]. Importantly, the sum of these gastrointestinal transformations and food matrix interactions can either increase or decrease the absorption of the resulting metabolites in the bloodstream.

### 3.3. Plasma Bioavailability, Transformations and Cellular Uptake

Once in the blood stream, enzymes in the liver and kidneys further modify polyphenols into various conjugated forms, a process that serves to detoxify potentially harmful substances. Molecules are rendered more hydrophilic in order to facilitate their urinary elimination, which usually lowers bioavailability [66,67]. While metabolites usually constitute the greatest fraction of circulating polyphenolic species, some forms undergo enterohepatic recirculation via biliary secretion, followed by deconjugation into free polyphenols by the gut microbiota and reabsorption in the colon [68,69,70]. Additional hepatic reactions may also occur which revert circulating metabolites back to the free form [71,72,73], as is the case for the conversion of resveratrol sulphate to bioactive resveratrol by sulphatases in humans [73]. Moreover, glucuronide and sulphate metabolites retain some of their beneficial effects in vitro [74,75]. Thus, chronic administration of polyphenols may be an efficient strategy to increase plasma bioavailability in humans, as reported for epigallocatechin-3-gallate (EGCG) [76].

The final step in the action of polyphenols is cellular uptake, which depends not only on how they have been metabolized but also on their interaction with circulating proteins, fatty acids and lipoproteins [77] with the bioefficacy of therapeutic agents heavily relying on binding to such serum transporters [78]. Resveratrol for example, is lipophilic which requires transformation into a more hydrophilic form, by sulphation, glucuronidation or binding to proteins enabling circulation in appropriate concentrations [79]. The formation of complexes between resveratrol and transporter proteins, principally albumin [80,81,82] and lipoproteins [83,84,85,86], impedes its uptake by cells [79]. Fatty acids are also known to improve the ability of resveratrol to bind transporter proteins [87].

While the binding by transporter proteins diminishes the availability of the free form of the polyphenol, it is thought to provide a polyphenol reservoir, important in the systemic distribution of bound species [77]. Some studies have proposed that these complexes are retained at the cell membrane by albumin and lipoprotein receptors, offering a carrier-mediated mechanism by which polyphenols may gain entry to cells [77] in addition to passive diffusion [79]. There is also the possibility that polyphenols need not enter cells to have an effect, as when free resveratrol binds integrin αVβ3 [88] to produce an angiosuppressive effect (Belleri et al., 2008) and when it triggers p53-dependent apoptosis of breast cancer cells [89].

### 3.4. Accumulation in the Brain Parenchyma

Drugs targeting the brain must ultimately be able to accumulate in the brain parenchyma, in a biologically active form and in sufficient concentrations. Three important obstacles stand in the way of this: the blood-brain barrier (BBB), efflux transporters and multidrug resistance-associated proteins [90,91]. Youdim and colleagues were the first to demonstrate polyphenols crossing the BBB in an in vitro model, describing superior penetration of lipophilic (methylated conjugates) in comparison to hydrophilic molecules (sulphated or glucuronidated) [92,93]. Another study identified a stereoselective process in the passage of flavonoid catechins across the BBB [94]. Yet, the exact mechanisms polyphenols use by to traverse the BBB in vivo, either via diffusion or via transporters, remains to be elucidated.

Although information on transport of polyphenols into the brain is limited compared to the measurement of plasma levels, an increasing number of studies have measured polyphenols and metabolites in the brains of rodents and pigs [95], as reviewed elsewhere [90,96,97]. Entry into the CNS of the most commonly studied polyphenols has been reported several times, for resveratrol [67,98,99,100,101], EGCG [102,103] and quercetin [93,104,105,106].

However, differences in uptake are reported depending on the route of administration and the methods used for measurement. For example, in one study, orally administered tritiated resveratrol in rats (50 mg/kg b.w.) was reported to reach 1.7% of the ingested dose in the plasma and below 0.1% in the brain after 2 h [67]. Interestingly, 18 h after administration, the CNS retained 43% of the resveratrol measured at 2 h, mainly in the free form. Despite this retention in the brain, resveratrol levels, measured by high-performance liquid chromatography (HPLC) are lower than in the liver, kidney, testes and lungs [99]. However, another study was unable to detect brain resveratrol or metabolites in rats fed a 0.2% resveratrol diet for 45 days using HPLC with a detection limit of 0.5 pmol/mL/mg [107]. Other studies have also used chromatographic methods to measure resveratrol in rat brains using different protocols. In one study, 15 mg/kg b.w. of resveratrol were administered intravenously (i.v.), a relatively high dose, with brain tissue concentrations reaching ~0.17 nmol/g after 90 min [99]. Another study administered escalating oral doses of resveratrol (100–400 mg/kg b.w.) for 3 days and detected ~1.7 nmol/g in the brain by liquid chromatography-mass spectrometry [100].

Some polyphenols are extensively transformed before they reach the brain, which may dampen their bioavailability, as discussed above. As an example, curcumin is highly lipophilic and, in theory, should easily gain entry to the brain [108]. However, before reaching the BBB, the free form of curcumin is rapidly conjugated, rendering it only sparingly bioavailable to the CNS [109]. Conversely, catechins efficiently cross the BBB after oral administration but are found in glucuronidated and 3′-*O*-methyl glucuronidated forms in the brain [102,110]. To date, it remains unclear whether conjugation occurs before or after entry into the brain. Nevertheless, strategies exist to boost CNS concentrations of the aglycone form, for example by continuous administration aimed at promoting tissue accumulation [103]. Following 24 h of continuous intragastric administration, EGCG levels in the CNS reached 5–10% of concentrations measured in the plasma [103]. These results imply, however, that a very high plasma concentration is needed for EGCG to accumulate in therapeutically reasonable concentrations in the brain. The necessity of maintaining high circulating concentrations may raise questions regarding the safety and tolerability of polyphenols.

### 3.5. Synergistic Effects

Some polyphenols interact beneficially when administered in combination. Synergistic pharmacokinetics are at the basis of emerging multi-drug therapies [111,112,113] developed to surmount problems of low efficacy, acquired resistance and undesirable side effects in standalone treatments. Polyphenols synergize via multiple mechanisms, extensively reviewed elsewhere [114,115,116]. Although synergistic chemosensitization properties of polyphenols are well known, for example EGCG-induced downregulation of endoplasmic reticulum stress response elements rendering temozolomide treatments more efficient in a mouse model of glioma [117], what follows will concentrate solely on neuroprotective mechanisms.

Underlying the efficacy of herb and plant extracts, different polyphenols may concurrently regulate the same or separate targets in cells, resulting in a concerted agonistic effect. For instance, combinations of resveratrol and quercetin [118,119] or epicatechin and quercetin [120] synergize to protect against amyloid-like aggregation, oxidative stress and oxygen-glucose deprivation in vitro. An earlier report of synergy between polyphenols showed that treatment of neuronal PC12 cells with suboptimal doses of resveratrol in combination with catechin conferred greater protection against Aβ toxicity than the sum of their individual actions [121]. However, when measuring their free radical scavenging activities, the authors found their combined antioxidative effect to be merely additive, suggesting that their synergistic neuroprotective competences at combined subliminal doses may depend on other cellular mechanisms [121]. Very few studies have addressed neuroprotective synergy in vivo though a combination of polyphenols was found to synergistically rescue photoreceptors in an animal model of retinal degeneration [122].

Synergy can also occur between polyphenols, drugs and hormones. Many in vitro reports support this, as is the case for the potentiation of neurite outgrowth by a subeffective dose of brain-derived neurotrophic factor (BDNF) in conjunction with green tea catechins [123,124], as well as the protection of primary neurons and astrocytes by a cocktail of suboptimal doses of resveratrol and melatonin via upregulation of heme oxygenase-1 [125]. One of the first reports of polyphenol-drug synergy in rodents showed EGCG favorably interacting with rasagiline, an irreversible inhibitor of dopamine-metabolizing monoamine oxidase B (MAO-B) for the treatment of Parkinson’s disease [126,127]. When administered alone in suboptimal doses, neither EGCG nor rasagiline were capable of rescuing nigrostriatal neurons in a 1,2,3,6-tetrahydropyridine (MPTP)-injured mouse model of Parkinson’s disease [128]. However, in combination these agents in low doses promoted the survival of the dopaminergic nigrostriatal pathway, demonstrating their synergistic effect. Interestingly, the ability of rasagiline to promote the expression of BDNF in concert with EGCG-induced induction of protein kinase C produced a sum agonistic effect converging at their downstream effector Akt/protein kinase B, thought to account for their neuroprotective action. Other examples of polyphenol-drug synergies exist for valproate and resveratrol in ischemic stroke [129] as well as for glatiramer acetate and EGCG in experimental autoimmune encephalomyelitis [130].

Many polyphenols readily regulate absorption in the gastrointestinal tract, clearance at the level of the kidneys and detoxification in the liver by modulating the activity of transport proteins or metabolic enzymes, which may improve their own oral availability. This property has potential for use in Parkinson’s disease by minimizing levodopa methylation in the liver by inhibiting human catechol-*O*-methyl transferase (COMT), thereby enhancing bioavailability of the drug [131]. Flavonoids are also known to be potent inhibitors of cytochrome P450 (CYP) enzymes [132,133] whose activity reduces polyphenol bioavailability. This potential to enhance bioavailability of metabolism-sensitive drugs constitutes a clear example of polyphenol synergy that may be relevant in human treatment.

## 4. Safety and Tolerability

In addition to favorable pharmacokinetics, polyphenols must be safe and well-tolerated in humans. Several investigations have already addressed safety and tolerability issues (see for review [134,135,136,137]). What follows is a summary of these findings.

### 4.1. Side Effects from Dosage and Chronicity

Virtually all investigations performed in humans using a wide array of polyphenol preparations found that they are safe and tolerable in the short- [138,139], medium- [46,140] and long-term [141,142,143]. Generally, side effects are uncommon and are mild and transient and include minor gastrointestinal problems and, more rarely, headaches, dizziness and rashes. In a phase II trial, 24 Alzheimer’s patients were administered 2 or 4 g of curcuminoids daily for 48 weeks and 3 withdrew due to minor gastrointestinal issues [143]. A study using a single 5 g/70 kg b.w. intake of resveratrol, representing 1/40 of the nephrotoxic dose and 1/4 of the highest dose reported to be safe in rats [144], did not show any serious adverse effects [138]. A great number of investigations have also addressed the safety of specific diets enriched in polyphenol-rich foods. Of particular interest, black cohosh, soy and red clover regimens aimed at reducing menopausal symptoms in women have proven to be safe, with occasional mild gastrointestinal issues, musculoskeletal and connective tissue troubles, as well as weight gain (see for review, Reference [134]).

### 4.2. Adverse Pharmacological Interactions

While a consensus has been reached on the safety and tolerability of polyphenols in most individuals, certain contexts preclude their use. Grapefruit juice is an example of the possible effects of polyphenols under specific conditions. Apigenin, naringenin, nobiletin and hesperetin in grapefruit juice potently inhibit the detoxifying enzymes, members of the CYP family, responsible for the metabolism of several prescription drugs [132,145,146,147,148]. Interestingly, enzymatic inhibition is apparently irreversible following the ingestion of 200–300 mL of juice, leading to increased drug bioavailability and toxicity for up to 24 h after intake. Medical professionals are now mindful of the risks of consuming grapefruit juice in individuals already taking antidepressants such as buspirone (Buspar) and sertraline (Zoloft), beta-blockers, anti-cancer agents, fexofenadine (Allegra) or certain statins (atorvastatin) among other drugs [149,150,151,152]. Several other adverse interactions exist between polyphenols and drugs [153,154] and have been extensively discussed elsewhere [136].

### 4.3. Tumorigenicity

As previously discussed, certain polyphenols, termed phytoestrogens, are biofunctional due to their resemblance to steroid hormones. Members of the flavonoid and stilbene subclasses indeed possess the capacity to bind ERs [155] and testosterone receptors [156], albeit with much lower affinities than endogenous ligands. Many studies find phytoestrogens to be safe with respect to incidences of cancers [157,158] and support their role in inhibiting aberrant cell proliferation [159,160,161,162,163,164,165]. Nevertheless, a few publications draw attention to the possible carcinogenic actions of some phytoestrogens that should not be ignored [166]. In particular, soy genistein and daidzein (0.001–10 µM) may stimulate the growth of malignant breast tumors, both in vitro and in vivo [166,167].

In the case of the stilbene resveratrol, studies confirm its ability to bind both ERs [168], however with 7000 times less affinity than estradiol [33]. Interestingly, its effects are apparent for select EREs regulated by ERα but not for EREs dependent on ERβ activation. Unlike other ERα agonists, resveratrol does not appear to provoke mammary or uterine tissue proliferation in rats [169] and even promotes neuronal differentiation in vitro [170]. In light of this, resveratrol’s favorable effects may in fact partially hinge on tissue-specific expression profiles of ERα and ERβ [171]. More recently, a study delineated the discriminatory ability of resveratrol to impede inflammation without promoting cell proliferation through pathway-selective ERα activation [172]. Crystallographic studies of the ligand-binding domain revealed resveratrol to bind in the opposite orientation to estradiol, which may be at the core of its pathway selectivity and its proven safety in humans [135], particularly with regard to carcinogenesis.

## 5. Clinical Progress

The therapeutic potential of polyphenols is clear from the overwhelming body of literature supporting their beneficial effects in countless preclinical disease settings (see for review [16,17]). Notwithstanding the weight of epidemiological, anecdotal and fundamental evidence, translation from bench-to-bedside has proven challenging despite relentless efforts to test polyphenols in human trials (see [90] for a review). Currently, only a single trial looking at polyphenols in neurodegenerative disease has reached phase III clinical testing [173]. In this randomized, double-blind, placebo-controlled parallel group study, disease progression will be assessed after 48 weeks of daily oral EGCG treatments in multiple system atrophy patients.

The example of a standardized Ginkgo biloba extract, rich in flavonoids, yielded particularly disappointing results with numerous failed phase I trials [106,174,175,176]. These studies addressed dementia prevention in large cohorts of healthy or mildly cognitively impaired elderly individuals administered oral Ginkgo biloba twice daily for several years [177] but no reduction in the incidence of cognitive decline or Alzheimer’s disease was found [178,179,180,181,182]. Other phase I and II clinical attempts have also been unsuccessful in confirming the putative positive effects of curcumin in Alzheimer’s disease patients [143,183]. The reasons behind these results may be due to preclinical models failing to fulfill their predictive purpose or clinical trials may simply be incapable of detecting the beneficial effects of polyphenols due to a flawed approach. What is important to keep in mind is that successful clinical trials are not common, on account of the inherent difficulty of translating applications between rodents and humans.

To address this, the required recruitment profile for testing Ginkgo biloba extracts was re-evaluated, yielding positive results in a new round of clinical trials, this time performed in full-blown Alzheimer’s disease and vascular dementia. These trials successfully uncovered the benefits of several months of a daily Ginkgo biloba treatment on cognition and neuropsychiatric symptoms [141,142]. Changing the endpoints and focusing on prefrontal dopaminergic functions in elderly humans with self-reported mild cognitive decline was another fruitful strategy to reveal the beneficial effects of Ginkgo biloba [184]. Nevertheless, the cholinesterase inhibitor rivastigmine, commercially known as Exelon, has been shown to be more efficient than Ginkgo biloba in treating Alzheimer’s disease and remains the drug of choice to ameliorate cognitive impairment in mild to moderate forms of the disease [185].

Several other phase I trials have been successful in confirming small positive effects in healthy individuals. A variety of polyphenols, including resveratrol, were found to increase cerebral blood flow without, however, improving cognitive performances in young adults, whether administered in a single dose [186,187,188] or chronically over 28 days [189]. However, other groups found that longer chronic interventions in elderly humans using either cocoa flavanols or resveratrol enhanced dentate gyrus-related cognitive functions [190] and hippocampal-related memory functions [191], respectively. In Alzheimer’s disease patients, resveratrol reached phase II trials on the basis of its modulatory role on neuroinflammation, cognitive decline and cerebrospinal fluid (CSF) levels of Aβ40 [192,193]. Following a twice-daily oral regime for one year, resveratrol and its metabolites were present in the CSF, validating its ability to cross the BBB in humans [192]. Despite its relatively low bioavailability, resveratrol remains a candidate for potential use in human neurodegenerative diseases.

## 6. Future Strategies for Pharmaceutical Development for Neuroprotection

Polyphenols have interesting properties that justify efforts to translate their potential neuroprotective effects into treatment for human neurodegenerative diseases. However, their questionable bioavailability, modest effects in humans and the impossibility of applying patent protection on natural molecules detracts from the appeal of polyphenols for pharmaceutical use. Nevertheless, several strategies have been used by drug development in recent years to tackle these issues.

### 6.1. Alternative Preparations and Prodrug Approches

The engineering of novel structural analogues inspired by existing polyphenols or formulating specific preparations of polyphenols, such as the well-defined Ginkgo Biloba extract 761, may be patentable options. Among the latest innovations, chemical engineering of pro-drug polyphenolic structures has shown promising results. For instance, acetylation of EGCG or resveratrol via esterification of their hydroxyl moieties yields stable pro-drugs in vivo whose acetyl groups can be hydrolyzed intracellularly by esterases to release the free polyphenol within the cell [194,195,196]. This strategy minimizes polyphenol auto-oxidation and allows better lipophilicity-dependent cellular uptake [197,198,199]. Production of conjugates with improved bioefficacy has also been a good approach to promote polyphenols absorption and activity. For example, the glutamoyl diester of curcumin is a more potent neuroprotective agent than curcumin [200] and similar approaches have been deployed for resveratrol [201,202]. More importantly, prodrugs of resveratrol are promising as recently reviewed in Biasutto et al. [203], for delivery to the brain parenchyma.

### 6.2. Alternative Drug Delivery Systems

Another favorable approach is the development of novel encapsulation technologies. Progress in vehicle formulation has allowed polyphenols to be contained in lipid nanocapsules [204,205,206], nanoparticles [206,207], exosomes [208], nanocomposites [209], emulsified formulations [206,210,211] or in gel form [212]. Several reports demonstrate increased bioavailability for encapsulated polyphenols in rodents [213,214]. Another unusual approach is the administration of biologically compatible carbon nanotubes [215] grafted with polyphenols, such as gallic acid [216]. This method was shown to enhance the antioxidative properties of grafted agents [216] and to improve their ability to traverse biological barriers [215,217], although the application of such conjugates is still not common, and the outcomes have not been sufficiently addressed. Possible health concerns of using carbon nanotubes also warrant further investigations [215,218]. Another simple tactic consists in improving solubility of polyphenols in circulation, such as for the lipophilic resveratrol [219], via coupling to cyclodextrins, which have the capacity to form inclusion complexes and this approach has already been exploited in other drug delivery strategies [220]. Overall, each of these methods has advantages and disadvantages but brain accessibility is generally augmented owing to improved BBB infiltration by lipophilic vehicles, brain targeting by encapsulation and blocking the metabolism of polyphenols [221].

### 6.3. Alternative Administration Routes

In order to target the human brain more efficiently, the route of administration is another variable that can be altered. The most promising of these is intranasal administration, usually paired with one of the previously described encapsulation techniques, which has proved successful for brain-targeted drugs in humans, at least for increased bioavailability and the avoidance peripheral side effects [222,223]. Notable examples are the administration of insulin for the treatment of Alzheimer’s disease [224] and apomorphine for the treatment of Parkinson’s disease [225]. The mechanisms by which drugs can be delivered to the brain parenchyma are only beginning to be explored. It would appear that drugs administered nasally either enter the brain through retrograde axonal transport at the level of the olfactory sensory cells or by penetration into the CSF across the nasal epithelium [226]. Although studies with polyphenols are scarce in preclinical models [227,228,229], intranasal curcumin administration has gained attention (see for review [230]) due to its very poor oral bioavailability [231] but promising neuroprotective actions. Curcumin is highly lipophilic and may easily cross the BBB [108] if it is delivered into the bloodstream and protected from enzymatic modifications [232]. While it is generally recognized as a safe route, intranasal administration sometimes leads to minor adverse effects, principally nasal irritation, constituting a potential problem in the development of intranasal polyphenol administration [225,233]. More unusual administration systems for polyphenols include rectal suppositories for efficient systemic distribution, bone-marrow administration for immunomodulatory effects and controlled-release implant strategies for targeting tumors. Intrathecal administration for direct distribution in the CSF of curcumin remains a favorable yet invasive option for brain targeting (see for review, Reference [230]).

## 7. On the Topic of Dose-Response

To prove that polyphenols can accumulate in high-enough concentrations in target tissues as the brain is linked to the antioxidative properties of polyphenols in vitro [234,235].

More recently, the physiological significance of the direct antioxidative actions of polyphenols is met with skepticism, particularly with regard to the action in the brain, due to limited gastrointestinal absorption, propensity to undergo biotransformation and rapid excretion by the kidneys [97,236]. On the one hand, H-atom transfer must always occur faster than at least one of the reactions of free-radical-production cascades (e.g., the limiting step in lipid peroxidation) and this is improbable [237]. On the other hand, polyphenol concentrations, which rarely exceed micromolar concentrations in plasma or tissues [238] are substantially inferior to those of endogenous antioxidants such as ascorbate (30–100 µM) and urate (140–200 µM) [239]. Consequently, it is argued that their contribution to the total antioxidative capacity of the plasma never exceeds 2% and may therefore be irrelevant in a physiological context [236,240]. In fact, direct antioxidative effects of polyphenols have not been measured in the brain [97]. Also, studies demonstrating the anti-inflammatory properties of polyphenol analogues, other than direct antioxidative actions, challenges the idea that their health effects stem from their ability to hamper oxidative stress [201,202].

Nowadays, it is acknowledged that high circulating concentrations of polyphenols may not be required to achieve certain clinical endpoints. Indeed, by interacting with various enzymatic targets, for instance Keap1, very small doses of polyphenols may benefit from the cascades of events that ensue in cells. Despite this, efforts continue to focus on enhancing bioavailability rather than on identifying an adequate dose-response framework that could predict the behavior of this class of molecules. This oversight may partly account for the apparent difficulty of translating preclinical findings into actual positive outcomes in humans. Where disappointingly modest clinical benefits have been shown, is increasing the dose always a judicious strategy? The answer may not be as obvious as once thought.

Explanations have been proposed to explain the bioefficacy of polyphenols at very low doses. One of these is that polyphenols exert their biological effects in a non-linear fashion by exhibiting a biphasic dose-response profile. One such model predicts J or inverted U dose-response curves depending on the endpoint [241,242]. The biphasic theory stipulates low-dose stimulatory and high-dose inhibitory effects [243,244]. It direct stimulatory effects at low concentrations followed by biological overcompensation at higher doses [245]. In neuroprotection, hormesis predicts very low doses as beneficial and higher doses as potentially harmful. The application of this theory is thus intimately linked with whether polyphenols are indeed stressors that induce a defense response in cells. This has yet to be confirmed for polyphenols.

At present, the biphasic hypothesis explaining the bioefficacy of polyphenols at very low doses is gaining momentum, resveratrol constituting the best example. A wealth of reports support the hormetic action of resveratrol in various applications, ranging from cancer to neuroscience, extensively reviewed elsewhere [246]. In some instances, resveratrol stimulates cancer cell proliferation at very low doses but inhibits carcinogenesis in higher concentrations [247]. Other reports show resveratrol inducing atherosclerotic lesions at high doses, while it remains cardioprotective at lower concentrations [248]. In neurons, resveratrol promotes survival at very low concentrations but is neurotoxic at higher doses [121,249]. One study performed in mice and primary cortical neurons proposed a mechanism possibly underlying the biphasic response of energy-depleted neurons to resveratrol, showing protection at low doses and toxicity at higher doses [250]. The authors explained resveratrol’s bimodal effects via its stimulatory action on silent mating type information regulation 2 homolog 1 (SIRT1), whose low-grade activity can suppress oxidative stress [251]. However, when stimulated by greater doses of resveratrol, SIRT1 expends too much-reduced nicotinamide adenine dinucleotide (NAD+) where neurons are already energetically depleted, causing energy failure. During an ischemic event, resveratrol administration could be either beneficial or detrimental, depending on dosage and timing and the bioenergetic status of neurons.

At present, these studies are usually performed in pre-clinical models and do not necessarily reflect what could occur in humans. The best-documented evidence of biphasic dose-responses in humans is for radiation, for instance in cancer treatments or in atomic bomb survivors [252,253]. However, reservations remain on the significance of such a dose-response relationship in the human brain, as it is highly unlikely that polyphenols could ever increase bioavailability in the parenchyma beyond low concentrations. This means that the observed bioefficacy of polyphenols may already be optimal where modest benefits are found in trials. Indeed, one distinct feature of the biphasic hypothesis provides that beneficial effects at low doses stem from cellular overcompensation mechanisms in response to the polyphenol-induced stress [254]. Beyond the optimal concentration at which maximal benefits are seen this compensation reaction is slowly overwhelmed by the increasing stress polyphenols directly exert on the cell. Even at the optimal concentration, these beneficial effects are thus thought to be at best partial. If this theory holds true, this could explain the results of clinical trials to date, even upon increasing dosages.

## 8. Concluding Remarks

The chemical structure of polyphenols confers them metal-chelating, fibril-destabilizing, estrogen-like, enzyme-binding and indirect antioxidative effects supporting their usefulness in neurodegenerative diseases. Epidemiological evidence shows a strong association between polyphenol consumption and reduced occurrence of various neurodegenerative diseases. Preclinical models lend them neuroprotective properties. Some clinical trials have even been successful in revealing small but measurable improvements in human health and have confirmed their safety in various settings. Nevertheless, the limited bioavailability of polyphenols together with their apparent bioefficacy remains under-explored. Investigators must demonstrate that polyphenols exert significant health benefits. However, in neurodegenerative diseases, polyphenol trials consistently fail in early clinical testing. To overcome this, researchers must optimize the design of their trials, subjects (disease stage, participant profile, cohort age and medical history), polyphenol administration (polyphenol formulation, route, dosage, frequency and duration) and endpoints (motor symptoms, cognitive decline, neuroinflammation, neuron integrity, CNS vascular health, etc.). As reviewed here, polyphenols are sensitive to a great number of physiological conditions that impinge on their bioavailability and biofunctionality, which may account for the markedly high inter individual variation observed in clinical investigations, which cannot be explained by biphasic dose-response theories.

Despite a large amount of information from many pre-clinical disease models and applications, a working theoretical framework that could aid in predicting outcomes in humans cannot be agreed. A priority would consist of determining the maximal health benefits that could be achieved from polyphenol monotherapies as they most usually stand alone in trials. Can we really expect standalone treatments to fulfill hard-to-reach clinical endpoints? If epidemiological evidence is strong for the protective effects of consuming complex mixtures of polyphenols in food, it may be unjustified to expect single molecules to be as effective. Perhaps concentrating on the concerted effects between polyphenols with each other or with other drugs that show partial benefits, such as the MAO-B inhibitor rasagiline [127] or levodopa [131], may overcome the as yet modest effects in humans. Evaluating polyphenols in preventive clinical paradigms may also constitute a more realistic strategy.

Besides, recent nutrigenomics data show that the interaction between genes and food bioactive compounds can positively or negatively influence an individual’s health and possibly will aid with the prescription of customized diets according to an individual’s genotype. Thus, the next approaches to clinical research with polyphenols should consider that dietary bioactive compounds such as polyphenols can be attributed to epigenetic mechanisms such as the regulation of histone deacetylases (HDAC) and histone acetyltransferase (HAT) activities and acetylation of histones and non-histone chromatin proteins [255,256].

## Abbreviations

Aβ	beta amyloid
AREs	uridylate (AU)-rich elements
ATP	adenosine triphosphate
BBB	blood-brain barrier
BDNF	brain-derived neurotrophic factor
COMT	catechol-*O*-methyl transferase
CNS	central nervous system
CSF	cerebrospinal fluid
cAMP	cyclic adenosine monophosphate
CYP	cytochrome P450
EGCG	epigallocatechin-3-gallate
ERs	estrogen receptors
ERα	estrogen receptor alpha
ERβ	estrogen receptor beta
ERE	estrogen response element
HPLC	high-performance liquid chromatography
Keap1/Nrf2/ARE	Kelch-like ECH-associated protein 1/nuclear factor erythroid 2-related factor 2/antioxidant response elements
MAO-B	monoamine oxidase B
NAD+	nicotinamide adenine dinucleotide
ArO•	polyphenolic radical
SIRT1	silent mating type information regulation 2 homolog 1
WBSSH	White–Bate-Smith–Swain–Haslam
MPTP	1,2,3,6-tetrahydropyridine
